# Psychological Fears among Low-Paid Female Sex Workers in Southwest China and Their Implications for HIV Prevention

**DOI:** 10.1371/journal.pone.0111012

**Published:** 2014-10-17

**Authors:** Shan Qiao, Xiaoming Li, Chen Zhang, Yuejiao Zhou, Zhiyong Shen, Zhenzhu Tang, Bonita Stanton

**Affiliations:** 1 Prevention Research Center, Carman and Ann Adams Department of Pediatrics, School of Medicine, Wayne State University, Detroit, Michigan, United States of America; 2 Institute of Global Health, Vanderbilt University, Nashville, Tennessee, United States of America; 3 Guangxi Center of Disease Control and Prevention, Guangxi, China; China Medical University, China

## Abstract

Commercial sex plays a critical role in rapidly increasing heterosexual transmission of HIV in China. Low-paid female sex workers (FSWs) are especially vulnerable to HIV/AIDS. Because of the illegality and stigma associated with sex work, FSWs may constantly live with fears in their daily life. Based on cross-sectional study of 794 low-paid FSWs in China we described their psychological fears related to commercial sex and examined the associations between fears and HIV-related behaviors. Fear of HIV infection was significantly associated with consistent use of condoms with clients. However, fear of breaching sex worker identity significantly prevented the FSWs from consistently using condoms with clients and taking HIV tests. Fear of being arrested by the police was positively associated with consistent use of condoms but negatively associated with accessing HIV prevention services. Our findings underlined the importance of examining the triadic interaction of behavioral, psychological and environmental factors in HIV prevention interventions among low-paid FSWs.

## Introduction

Compared with other women of reproductive age, female sex workers (FSWs) are taking a disproportionately high burden of HIV infection due to biological, behavioral and structural risk factors [1]. According to a recent meta-analysis among FSWs in low- and middle-income countries, the overall HIV prevalence is 11.8% with a pooled odds ratio of 13.5 for HIV infection [1]. Among this high-risk population, low-paid FSWs were especially vulnerable and marginalized due to their social-demographic characteristics [2], life history (e.g. child sexual abuse, adult sexual assault) [3], and working environment [4]. Low-paid FSWs refer to the FSWs who usually work on the street or lower-tier commercial sex venues and charge much less for each sex trade [5]. Struggling at the low end of the commercial sex hierarchy, they were fragile in both physical and psychological well-beings with problems of partner violence (e.g., physical or sexual abuse) [6], substance abuse (e.g., drug or alcohol dependence) [3], [7], depression and posttraumatic stress disorder symptoms [8], [9], and poor quality of life [10].

Existing studies also found that low-paid FSWs face a high risk of sexually transmitted infections (STIs) [11], [12] and HIV infection [13]. They may be also more likely to engage in unprotected sex than their high-paid counterparts [14], [15]. The HIV-related behaviors among this high-risk population may be influenced by socioeconomic factors and the contexts of their lives [16]. In Italy, some low-paid FSWs may not insist using condoms due to fear of losing clients or desire of getting extra pay for unprotected sex [17]. In Bangladesh, some low-paid FSWs spend very little money for health purposes, which may hinder their access to STI diagnosis and treatment [11]. A study among low-paid FSWs in India reported sexual coercion and forced group sex in the context of alcohol use, which made condom use negotiation difficult or even impossible [6]. In addition, drug dependence was associated with elevated rates of injection drug use and sexual risk behaviors among low-paid FSWs [18].

Commercial sex is illegal in China, but it has thrived since the economy reform in the late 1970s due to rapidly widening income disparities, increasing population mobility, and changing norms of sexuality [19]. It has also been driven by surplus rural labor and limited employment opportunities for women during the past three decades [20]. An estimated 4–10 million women are engaged in commercial sex industry with various categories [2]. According to national HIV sentinel surveillance data, the HIV prevalence rate was 0.3% among FSWs in China [21]. Although HIV prevalence rate among FSWs in China has remained relatively low in general, it is as high as 16% in some areas with dual epidemics of drug and HIV [20]. A serial cross-sectional study conducted between 2008 and 2012 suggested a decreasing trend of HIV prevalence (from 0.6% to 0.3%) but an increasing trend of syphilis prevalence (from 2.4% to 3.2%) [22]. In addition, there was a disparity between high-paid and low-paid sex workers in both disease prevalence burden in each year and the overall trends during the study period. HIV prevalence rates were higher among low-paid sex workers in both 2008 and 2012. There was a non-significant upward trend among lower-paid FSWs although syphilis prevalence rate was significantly reduced among higher-paid FSWs [22].

Low-paid FSWs in China are more likely to have higher prevalence of HIV infection and STIs, such as syphilis, HSV-2, and gonorrhea than their high-paid counterparts [23]–[25]. For instance, a recent systematic review reported a 2-fold increased risk of syphilis among low-paid FSWs [26]. Compared with high-paid FSWs, low-paid FSWs were older, less educated, and more often divorced or widowed [27]. Their vulnerable socio-demographic characteristics may contribute to their higher rates of unprotected sex [27], and lower utilization of HIV prevention services [27], [28].

Although recent research on FSWs in China has started to pay attention to the heterogeneity of China's commercial sex industry and attempted to focus on low-paid FSWs, few studies have explored the role of psychosocial factors on HIV-related behaviors in this group [19]. Due to illegal, clandestine and discriminatory nature of commercial sex in China, FSWs may constantly live with psychological fears which can lead to a number of psychological problems such as stress, depression, hopeless, pessimism in their daily life [29]. The negative emotions were shown to be associated with high risk sexual behaviors [29]. For example, Hong and colleagues reported that depression was associated with FSWs' inconsistent use of condoms with their clients [8]. Nevertheless, empirical data are still limited regarding psychological fears in the context of commercial sex among low-paid FSWs, and the relationship between their fears and HIV-related behaviors (consistently using condoms, participating in interventions activities, and HIV-testing).

Fear is defined as “the unpleasant emotional state consisting of psychological and psychophysicological responses to a real eternal threat or danger, including agitation, alertness, tension, and mobilization of the alarm reaction” [30]. Fear is often caused by perceived threat which is composed of perceived susceptibility (likelihood of personally experiencing the threat) and perceived severity (magnitude of harm from the threat) [31]. Several meta-analysis studies have suggested that the stronger the fear, the greater the attitude, intention, and action of behavior change [32]–[34]. However, there is scarcity of elaboration on the role of psychological fears among low-paid FSWs. What they are afraid of may reflect their working and living contexts and interplay with their behaviors. Studies on fears therefore may demonstrate complex triadic interactions of socio- structural factors (policy, legislation and social stigma), psychological factors and HIV-related behaviors.

Utilizing data from China, the current study will explore the issue of fears among low-paid FSWs with three specific objectives: First, to delineate content of fears among low-paid FSWs; Second, to explore how the content of fears may vary along with different demographic factors, work-related factors and perceptions related to HIV/AIDS; Third, to examine associations between fears among this vulnerable population and their HIV-related behaviors. To achieve the third objective, we will test three hypotheses: 1) fear of HIV infection and fear of STIs may be positively associated with FSWs' consistent use of condoms with clients; 2) fear of police arrests and/or fear of breaching their sex worker identity may be psychological barriers for HIV testing; and 3) fear of police arrests and/or fear of identity breaching may also prevent low-paid FSWs from accessing HIV prevention services.

## Methods

### Study site and survey procedure

Quantitative data were collected in 2011 from a tourist city in Guangxi Zhuang Autonomous Region (Guangxi). Guangxi is ranked second at provincial level in terms of HIV prevalence and first in terms of newly reported HIV infection cases in China [35]. Based on the national sentinel surveillance data in 2010, the overall prevalence of HIV, syphilis, and HCV infections among FSWs in Guangxi were 1.0, 6.1, and 1.0%, respectively [36]. Located in the northeast of Guangxi with a population of 1.34 million, the study city is famous for tourism attracting as many as 10 million visitors each year. Commercial sex has grown rapidly along with the flourish of tourism in this city where an estimate of 2,000 FSWs worked in at least 150 commercial sex venues [28].

With the assistance of local health workers, the research team conducted ethnographic mapping for various types of low-tier commercial sex venues/locations in the city. These venues included small roadside restaurants, mini-hotels, hair salons, feet-massage salons, streets (e.g., parks, city squares, and road-side), and other small entertainment establishments (e.g., karaokes and night clubs). The research team then contacted the owners/managers or other appropriate gatekeepers of these venues for their permission to conduct survey in their premises. Once we obtained permission from the gatekeepers, trained outreach health workers from the local Center for Disease Control and Prevention (CDC) approached the FSWs in the venues to ask for their participation. The street-based FSWs were recruited by direct contact and peer referral. Around 75% of the venue owners or managers and 70% of the FSWs approached agreed to participate in the survey. A total of 794 FSWs from 131 venues/locations provided written informed consent and completed a self-administered survey on their demographic information, fears related to their work, as well as HIV/AIDS related knowledge, attitudes and behaviors.

The paper survey was conducted in separate rooms or private spaces in the venues/locations to ensure confidentiality of the responses. No one was allowed to stay with the participant during the survey except the interviewer who provided the participant with necessary assistance. The questionnaire took about 15 minutes to complete. The study protocol was approved by the Institutional Review Board at Guangxi CDC in China.

### Measures

#### Demographic characteristics

Participants were asked about their age, ethnicity, marital status, home residence, education attainment, number of people in the family, and annual household income. For the purpose of data analysis in the current study, we dichotomized ethnicity into Han and non-Han, marital status into ever married and never married, and education attainment into less than middle school versus at least middle school.

#### Work-related factors

FSWs were asked to provide information on their work-related conditions and context, including length of working in commercial sex industry, types of work venues, age groups of most of their clients, number of clients per week, fees charged for each trade, income from sex work in past month, experience of police arrest, and experience of drug use. In the current study, drug use was defined to have ever used any type of illicit drugs including heroin, cocaine, marijuana, opioids, pethidine, ketamine, and ecstasy.

#### HIV awareness

To assess the HIV awareness, the participants were asked how much they knew about HIV/AIDS with a 4-point response (“not at all”, “a little”, “some”, “a lot”). Participants answered “some” or “a lot” were considered as having relatively high level of HIV/AIDS awareness.

#### HIV susceptibility

The FSWs were asked to assess the possibility they might get HIV infection using a 4-point scale from “very impossible” to “very possible”. For the purpose of data analysis in this study, responses of “very possible” and “possible” were coded as “high HIV susceptibility”, while “impossible” and “very impossible” were coded as “low HIV susceptibility”.

#### Psychological fears

To assess FSWs' fears in the context of commercial sex, we employed a multiple-response question “what are your fears when you are working in commercial sex”. The potential answers included “HIV infection”, “STIs”, “not getting paid by clients”, “being arrested by the police”, “family members become aware of the nature of my job”, “acquaintances become aware of the nature of my job” and “others”. In data analysis, fear of family members and fear of acquaintances becoming aware of their work were combined into a single item of fear of breaching their sex worker identity.

#### Consistent use of condoms with clients

The participants were asked about the frequency of using condoms with clients in the past month. The potential answers were “never”, “sometimes” and “every time”. The respondents who answered “every time” were considered to consistently use condoms with clients in past month.

#### HIV testing and HIV prevention service utilization

The participants were asked whether they had tested for HIV in the past year. They were also asked in the past year if the FSWs had participated in any of the three HIV prevention activities including “condom distribution and/or voluntary counseling and testing (VCT)”, “community-based methadone maintenance treatment program and/or needle exchange program”, and “peer HIV/AIDS education”. The FSWs who reported using at least one of these HIV prevention services were viewed as “ever used HIV prevention services”, and otherwise they were considered as “never used HIV prevention services”.

### Data analysis

First of all, we conducted descriptive analysis to illustrate the content of fear among the FSWs. Specifically, responses to the question “what are your fears when you are working in commercial sex” were transferred into seven binary variables indicating whether or not a specific fear was reported (Yes = 1, No = 0). The frequency and proportion of each response were calculated to demonstrate the levels of various fears among the FSWs. Second, we explored the relationship between each specific fear and the FSWs' background variables (demographic characteristics, work-related factors, and HIV-related perceptions). We employed one-way ANOVA (for continuous measures) and Chi-square test (for categorical measures) to compare the differences between groups in terms of background variables. Third, univariate and multivariate logistic regression models were employed to identify background variables that were associated with each HIV-related behavior (i.e., consistent use of condoms with clients, HIV testing, and utilization of HIV prevention service). Those background variables associated with the dependent variable at the p<0.2 level in univariate regression models were then entered into the multivariate logistic regression models. Three multivariate logistic regression models were conducted to examine the association between fears and each of HIV-related behaviors after adjusting background variables.

In second and third steps of the analysis, the variable of venue types was dichotomized into the venues with less mobility (feet-massage salons and hair salons) and the ones with more mobility (roadside restaurants, mini-hotels, street-based and other sites). In logistic regression analysis, two income variables were transformed by taking natural logarithms of their values because of their skewed distributions. We included four most commonly reported fears (i.e., fear of HIV infection, fear of STIs, fear of identity breaching, and fear of police arrests) in final multivariate logistic regression models. VIF (i.e., Variance Inflation Factor) test in the multivariate logistics regression analysis was thus employed to test potential collinearity between the four fear variables. All statistical analyses were performed using SPSS 16.0 (SPSS Inc, Chicago, IL).

## Results

### Background characteristics

The background characteristics and content of fears among the FSWs is presented in Table 1. The FSWs in the current study were 31 years old on average (SD = 8.9) and 85% of them were of Han ethnicity. Nearly 71% of the participants were Guangxi residents rather than migrants from other provinces. The majority of the FSWs were ever married, 41% had not completed middle school education. There were four members in each family on average. The mean annual household income was 14,195 yuan (SD = 12,132.9).

**Table 1:** Summary of background characteristics among low-paid FSWs in China.

N = 794	Frequency (%)	Mean (SD)
**Demographic factors**		
Age in years		31.1(8.9)
Ever married*	506(64.1%)	
Guangxi residence*	558(70.6%)	
Han-Ethnicity	676(85.1%)	
Education < middle school	324(40.8%)	
No. of people in household		4.0(4.2)
Annual household income		14195.0(12132.9)
**Work-related factors**		
Length of working as sex workers ≥1year	336(42.3%)	
Venue types		
Feet massage salon	75(9.40%)	
Hair salon	332(41.8%)	
Roadside Restaurant	137(17.2%)	
Mini-hotel	71(9.0%)	
Other establishment-based venues	63(7.9%))	
Street-based	116(14.6%)	
Age group of clients*		
<30	47(5.9%)	
30–50	635(80.3%)	
>50	109(13.8%)	
Charge per service (Yuan)		39.6(17.8)
No. of clients per week		18.9(2.7)
Income from sex work in past month (Yuan)		1911.9(1254.2)
Ever using drug	26(3.3%)	
Eve being arrested by the police	45(5.7%)	
**HIV-related perceptions**		
High HIV awareness*	502(63.3%)	
Low HIV susceptibility	638(80.4%)	
**Content of psychological fears**		
Fear of HIV infection	482(60.7%)	
Fear of STIs	403(50.8%)	
Fear of breaching sex worker identity	356(47.7%)	
Fear of being arrested by the police	317(39.9%)	
Fear of not getting paid by clients	103(13.0%)	
Other fears	8(1.4%)	

*Missing data was not included when we calculated the proportions.

About a half of the FSWs (51%) worked in salons (feet-massage salons and hair salons), 17% worked in roadside restaurants,15% were street-based, 9% worked in mini-hotels, and nearly 8% worked in other venues. Nearly 42% of the participants engaged in commercial sex for more than one year. About 80% of the FSWs reported that their clients were mainly aged 30–50 years, and 14% reported that the majority of their clients were older than 50 years. On average, the FSWs charged about 40 yuan for each sexual trade and had about 19 clients per week. The mean income from sex work in past month was about 1,912 yuan. Over 60% of the women reported that they knew some or a lot about HIV/AIDS, but around 80% perceived a low HIV susceptibility. Among all the respondents, 3.3% reported had ever used drug and 5.7% had ever been arrested by the police. Nearly a half of the FSWs had ever tested for HIV, and almost 73% had participated in at least one type of HIV prevention activities including condom provision/VCT, needle exchange, and peer HIV/AIDS education. However, less than 39% of the FSWs reported consistent use of condoms with clients in past month.

### Psychological fears among the FSWs

Most of the FSWs feared being infected by HIV (61%) or other STIs (51%). Nearly 48% of them worried about breaching their sex worker identity (either to family members or acquaintances). About 40% of the participants feared being arrested by the police. Some of the participants (13%) expressed their fear of not getting paid by clients. Only 1.4% of the FSWs reported fears other than those listed.

Table 2 illustrated the correlations between background characteristics and each fear variable. The FSWs who expressed the fear of HIV infection had a higher proportion of ethnic minority women, higher level of education attainment, and higher annual household income. As for work-related factors, more FSWs expressing the fear of HIV infection engaged in commercial sex for at least one year, and had higher income from sex work in past month. In addition, they were less likely to work in venues with less mobility and had a higher proportion of younger clients. The respondents who feared HIV infection also showed a higher proportion of ever using drug. In addition, HIV awareness and HIV susceptibility were higher among the FSWs who feared HIV infection than those who did not.

**Table 2:** Correlations between fear and background characteristics among low-paid FSWs in China.

	Fear of HIV infection		Fear of STIs		Fear of breaching FSW identity		Fear of being arrested by the police	
	No	Yes	No	Yes	No	Yes	No	Yes
**Demographic factors**								
Age in years (Mean, SD)	31.5(9.0)	30.8(8.8)	31.1(9.3)	31.1(8.5)	31.7(9.5)	30.3(8.1)*	30.7(8.7)	31.9(9.2)
Ever married	62.1%	65.3%	59.7%	68.2%*	63.7%	64.4%	61.9%	67.2%
Guangxi residence	70.0%	71.0%	71.6%	69.7%	73.1%	68.0%	72.4%	68.0%
Han-Ethnicity	88.8%	82.8%*	90.3%	80.1%***	85.8%	84.4%	85.1%	85.2%
Education < middle school	62.5%	57.1%*	37.1%	44.4%*	58.6%	59.9%	63.1%	53.3%**
No. of people in household (Mean, SD)	4.2(4.1)	4.0(4.4)	3.9(3.0)	4.2(5.1)	4.6(4.9)	3.5(3.2)***	4.3(4.5)	3.6(3.9)
Annual household income(Yuan, Mean, SD)	11741.6 (9929.9)	15791.0 (13138.6)***	11421.9 (10829.3)	16843.6 (12718.6)***	12228.4 (11551.7)	16361.9 (12401.3)*	12072.7 (11339.4)	17422.8 (12598.2)*
**Work-related factors**								
Length of working as sex workers ≥1year	36.9%	45.9%***	37.1%	47.4%**	41.4%	43.3%	36.7%	50.8%***
Working in venues with less mobility (feet massage salons and hair salons)	62.0%	48.1%***	50.6%	53.1%	36.4%	47.0%**	55.8%	46.1%**
Age group of clients (Year)								
<30	3.2%	7.7%	4.6%	7.2%	6.6%	5.3%	6.9%	4.4%
30-50	78.3%	81.5%	78.4%	82.1%	80.8%	79.7%	82.7%	76.6%
>50	18.4%	10.8%**	17.0%	10.7%*	12.6%	15.0%	10.3%	19.0%**
Charge per service (Yuan, Mean, SD)	38.1(18.7)	40.6(17.1)	39.9(22.0)	39.4(12.3)	36.9(21.0)	42.6(12.7)*	38.1(20.4)	41.8(12.5)*
No.of clients per week (Mean, SD)	19.2(2.5)	18.7(2.8)	18.7(2.7)	19.1(2.6)	18.7(2.7)	19.1(2.6)*	19.1(2.6)	18.6(2.7)*
income from sex work past month (Yuan, Mean, SD)	1688.8 (872.7)	2056.3 (1431.0)**	1642.2 (1065.4)	2173.5 (1364.7)***	1549.2 (887.5)	2309.1 (1461.1)***	1795.1 (1001.4)	2087.6 (1544.5)**
Ever using drug	1.0%	4.8%**	2.8%	3.7%	4.8%	1.6%***	1.7%	5.7%
Eve being arrested by the police	4.5%	6.5%	6.5%	5.0%	7.6%	3.7%*	5.5%	6.0%
**HIV-related perceptions**								
High HIV awareness	56.0%	66.0%*	64.4%	62.3%	65.8%	60.6%	61.6%	65.8%
Low HIV susceptibility	89.4%	77.0%***	87.7%	73.2%***	79.0%	81.8%	76.1%	86.8%***

*p<.05, **p<.01, ***p<.001.

Fear of STIs had similar correlations with background characteristics. The FSWs who feared STIs had a higher proportion of being married, being ethnic minority, with lower level of education attainment, and higher annual household income. More FSWs reporting the fear of STIs engaged in commercial sex for at least one year, and had younger clients. In addition, the fear of STIs was positively correlated with HIV susceptibility.

Fear of breaching sex worker identity was correlated with demographic factors, work-related contexts and experience but not with HIV awareness or HIV susceptibility. The FSWs with the fear of breaching their identity to others were younger, from smaller size families, with higher annual household income. More FSWs in this group worked in venues with less mobility (e.g., salons), had more clients each week, charged more for each service and had higher income from sex work in past month. Both the proportions of ever using drug and ever experiencing police arrests were significantly lower among the FSWs who feared breaching their identity compared with the FSWs who did not.

The FSWs who feared police arrests had higher level of education attainment and annual household income. A higher proportion of them worked in venues with less mobility, engaged in commercial sex longer, charged more for each sex trade, had older clients, with fewer clients each week and higher income from sex work in past month. The respondents who expressed the fear of police arrests reported lower perceived HIV susceptibility.

### Association between psychological fears and HIV-related behaviors

Table 3 and Table 4 show results of a series of univariate and multivariate logistic regression models to identify factors associated with HIV-related behaviors, respectively. Fear of HIV infection was significantly associated with consistent use of condoms with clients in past month (aOR = 1.88, 95% CI = 1.22, 2.91) after controlling other background characteristics and other fear variables. However, fear of STIs was not significantly associated with any HIV-related behaviors. Final logistic regression model indicated that fear of breaching sex worker identity significantly prevented the FSWs from consistently using condoms with clients (aOR = .66, 95%CI = .43,.99) and taking HIV tests (aOR = .48, 95% CI = .36,.73). As we expected, fear of being arrested by the police might hamper the FSWs' accessing HIV prevention service (aOR = .39, 95% CI = .22,.68). In addition, fear of police arrests was positively associated with consistent use of condoms (aOR = 1.60, 95% CI = 1.04, 2.45).

**Table 3:** Summary of univariate logistic regression models results.

	Consistent use of condoms with clients			HIV testing			HIV prevention service		
	OR	95% CI	p-value	OR	95% CI	p-value	OR	95% CI	p-value
**Demographic factors**									
Age	.933***	**[.914,.952]**	**.000**	1.003	[.986,1.020]	.745	1.017	[.998,1.037]	.079
Ever married	.563***	**[.411,.745]**	**.000**	1.085	[.811,1.451]	.581	**1.384** *	[1.005,1.908]	.047
Guangxi residence	.930	[.670,1.254]	.587	.950	[.699,1.290]	.741	.863	[.610,1.223]	.408
Han-Ethnicity	1.105	[.736,1.658]	.630	1.410	[.951,2.092]	.087	**1.582** *	[1.045,2.394]	.030
Education < middle school	1.876***	**[1.389,2.533]**	**.000**	1.100	[.830,1.462]	.504	.986	[.717,1.357]	.929
No. of people in household	.972	[.931,1.014]	.187	1.046	[1.000,1.095]	.049	1.026	[.978,1.077]	.297
Annual household income	1.905***	**[1.562, 2.324]**	**.000**	1.085	[.912,1.290]	.356	1.528***	**[1.242,1.878]**	**.000**
**Work-related factors**									
Length of working as sex worker ≥1 year	1.088	[.815,1.453]	.567	1.050	[.791,1.390]	.740	1.736**	**[1.266,2.381]**	**.001**
Working in venues with less mobility	**1.412** *	**[1.056,1.884]**	**.019**	1.800***	**[1.359,2.386]**	**.000**	1.736**	[.983,2.856]	
Age group of clients	.606**	**[.431,.853]**	**.004**	.514***	**[.367,.718]**	**.000**	.691*	[.484,.987]	.042
Charge per service	1.026***	**[1.015,1.038]**	**.000**	.998	[.990,1.006]	.561	**.989** *	**[.980,.998]**	**.021**
No.of clients per week	.821***	**[.768,.877]**	**.000**	.971	[.917,1.029]	.324	1.050	[.980,1.125]	.164
income from sex work past month	.915	[.776,1.079]	.290	1.686***	[1.404,2.024]	.000	2.072***	[1.714,2.504]	.000
Ever using drug	**2.650** *	**[1.187,5.918]**	**.017**	**.424** *	[.182,.987]	.047	.500	[.226,1.106]	.087
Ever being arrested by the police	.777	[.411,1.469]	.437	3.704***	**[1.808,7.590]**	**.000**	5.648***	[1.732,18.418]	.004
**HIV-related perceptions**									
High HIV awareness	2.060***	**[1.509,2.813]**	**.000**	3.636***	**[2.675,4.943]**	**.000**	6.029***	**[4.290,8.473]**	**.000**
Low HIV susceptibility	2.238***	**[1.503,3.333]**	**.000**	1.663**	[1.165,2.373]	.005	2.449***	[1.698,3.532]	**.000**
**Fear of**									
HIV infection	**1.976** *	**[1.458, 2.678]**	**.000**	1.250	[.940,1.663]	.125	1.194	[.870,1.640]	.273
STIs	1.125	[.844,1.498]	.421	.950	[.719,1.255]	.719	1.056	[.773,1.443]	.733
Breaching FSW identity	.994	[.746, 1.324]	.968	.475***	**[.358,.631]**	**.000**	.767	[.561,1.049]	.097
Being arrested by the police	1.952***	**[1.456, 2.615]**	**.000**	.655**	**[.492,.871]**	**.004**	.573**	**[.418,.786]**	**.001**

*p<.05, **p<.01, ***p<.001.

**Table 4:** Summary of multivariate logistic regression models results.

	Consistent use of condoms with clients			HIV testing			HIV prevention service		
	aOR	95% CI	p-value	aOR	95% CI	p-value	aOR	95% CI	p-value
**Demographic factors**									
Age	.954**	**[.926,.983]**	**.002**	-	-	-	1.033	[.993,1.076]	.111
Ever married	.749	[.486,1.156]	.749	-	-	-	1.455	[.835,2.533]	.185
Guangxi residence	-	-	-	-	-	-	-	-	-
Han-Ethnicity	-	-	-	1.009	[.644,1.581]	.989	.670	[.334,1.342]	.258
Education < middle school	1.177	[.757,1.830]	.470	-	-		-	-	-
No. of people in household	.989	.925,1.056	.734	1.015	[.977,1.054]	.442	-	-	-
Annual household income	1.771***	**[1.333, 2.353]**	**.000**	1.120	[.902,1.390]	.307	1.874***	**[1.339,2.621]**	**.000**
**Work-related factors**									
Length of working as sex worker ≥1 year	-	-	-	-	-	-	2.090**	**[1.218,3.585]**	**.007**
Working in venues with less mobility	.980	[.651,1.477]	.925	1.750**	**[1.241,2.467]**	**.001**	1.676	[.983,2.856]	.058
Age group of clients	.971	[.582,1.620]	.909	**.662** *	**[.441,.993]**	**.046**	.685	[.354,1.325]	.262
Charge per service	1.002	[.992,1.012]	.685	-	-	-	.983**	**[.971,.995]**	**.007**
No. of clients per week	.888***	**[.813,.971]**	**.009**	-	-	-	.939	.839,1.051	.272
income from sex work past month	.922	.686,1.239	.589	.956	[.730,1.251]	.741	1.537	[.967,2.443]	.069
Ever using drug	-	-	-	.607	[.224,1.640]	.325	.908	.263,3.136	.879
Ever being arrested by the police	-	-	-	2.748**	**[1.217,6.206]**	**.015**	4.585	.903,23.283	.066
**HIV-related perceptions**									
High HIV awareness	1.641*	**[1.069,2.519]**	**.023**	2.815***	**[1.964,4.036]**	**.000**	6.867**	**[3.974,11.867]**	**.000**
Low HIV susceptibility	1.320	[.777,2.242]	.304	1.143	[.736,1.776]	.552	.991	[.518,1.896]	.979
**Fear of**									
HIV infection	1.884**	**[1.218, 2.914]**	**.004**	1.271	[.883,1.828]	.197	1.015	[.561,1.836]	.962
STIs	.782	[.501,1.219]	.278	1.122	[.781,1.612]	.534	1.383	[.781,2.449]	.267
Breaching FSW identity	.661	[.433,.999]	.491	.514***	**[.363,.729]**	**.000**	.604	[.347,1.052]	.075
Being arrested by the police	**1.597** *	**[1.042, 2.447]**	**.032**	.785	[.549,1.122]	.183	.386***	**[.219,.681]**	**.001**

*p<.05, **p<.01, ***p<.001.

The VIF values for all the fear variables in the final model for each dependent variable (i.e., consistent use of condoms with clients, HIV testing, utilization of HIV prevention services) were less than 1.5 which was substantially smaller than the threshold value (e.g., VIF >10) that would suggest multicollinearity among the predictors in the model [37].

### Association between background characteristics and HIV-related behaviors

Some background characteristics were also independently predictive of HIV-related behaviors among the FSWs. Younger age, higher annual household income, and fewer clients each week were associated with FSWs' consistent use of condoms with their clients. Logistic regression models indicated that working in venues with less mobility, younger clientele, and high HIV awareness were positively associated with ever taking an HIV test. In addition, ever being arrested by the police was also significantly associated with having an HIV test before.

Finally, our data analysis suggested that FSWs who reported higher annual household income were more likely to participate in some HIV prevention service. Several work-related factors were also significantly associated with their utilization of HIV prevention service. The longer the FSWs engaged in commercial sex, the more likely they reported participation in HIV prevention service. Having a higher HIV awareness was positively associated with using HIV prevention service among the FSWs. The average charge for each sex trade was negatively associated with the FSWs' utilization of HIV prevention service.

## Discussion

To the best of our knowledge, the current study is one of the first quantitative studies to delineate content of fears among low-paid FSWs in China. Fear of HIV infection, fear of STIs, fear of breaching their sex worker identity, and fear of being arrested by the police were the most commonly reported fears among this high-risk population. Our findings indicate complex associations between fears and background characteristics as well as unique associations between different fears and various HIV-related behaviors.

It is interesting that the FSWs who expressed various fears were not always the most disadvantageous groups in terms of socio-demographic characteristics. We noticed that these FSWs reported higher annual household income and higher income from sex work in past month. Among the FSWs who feared HIV infection and police arrest, larger proportion reported higher level of education attainment. The FSWs who feared breaching their sex worker identity reported higher fees for each service. These results suggest that psychological fears might be more relevant or become more concerning among FSWs who were relatively better off in their lives. It is also notable that ever using drug correlated with fear of HIV infection. One potential explanation is that some FSWs who had ever used drug realized their high risk behaviors and perceived high susceptibility of HIV infection and severity of the threat (fear).

The associations of psychological fears with HIV-related behaviors may depend on content of fears and may also vary by specific behaviors. The relationship between fear of HIV infection and condom use with clients is consistent with existing theoretical and empirical studies on psychological fears. The more severity of threat people have realized, the more likely they are motivated to change behaviors in order to protect themselves [34]. However, fear of STIs was not significantly associated with any of the HIV-related behavior based on our data. One possible interpretation of this result is that the perceived severity of STIs was much lower than the one of HIV infection among low-paid FSWs. STIs might have been viewed as treatable and curable, while HIV infection was generally perceived as fatal. Further studies are needed to examine the differential patterns of relationships between fear of STIs and HIV-related behaviors.

Our study indicated that FSWs' fear of breaching their identity might be an important issue that needs to be addressed in HIV prevention. Because of the stigma against sex work many FSWs conceal the nature of their work to their families, friends, and acquaintances [38]. Fear of breaching their identity was negatively associated with consistent use of condoms because FSWs might worry about potential questioning or suspicions about the nature of their work when family members or acquaintances discover their carrying condoms with themselves. Fear of breaching their sex worker identity might prevent FSWs from accessing HIV-testing, which was also evidenced in existing studies on HIV-testing behaviors among FSWs in China [28], [39], [40]. Visiting HIV-testing facilities may increase the risks of breaching their sex worker identity as the concern of confidentiality of testing was one of major barriers for HIV testing among FSWs [28].

Commercial sex is illegal in China. The consequences of being arrested by the police include possible jail time, a large fine, and a breach of sex worker identity [5]. Fear of police arrests reflects an individual psychological response to a structural factor. Our study suggested that the FSWs who consistently used condoms with clients were more likely to express their fear of being arrested by the police. That may be because condoms at workplace were often taken by police as material evidence of commercial sex [5]. In addition, local police may compulsorily carry out HIV-testing among arrested FSWs as part of law enforcement, which may explain why the FSWs who had ever been arrested showed a higher proportion of HIV testings or using HIV prevention service.

Findings of the current study should be interpreted with caution due to several limitations. First, our sample may not be representative of low-paid FSWs in other areas of China. Our study was conducted in a tourist city of a multi-ethnic region. Most of participants were local residents rather than rural-to-urban migrants. Second, because of cross-sectional study data, the associations between fear and HIV-related behaviors may not be interpreted as causal. For example, fear of HIV infection may stimulate motivation to practice preventive behaviors; preventive behaviors may also shape perceptions of threat and further change fear [41]. The potential reciprocal relationship between fear and behavior merits further studies with a prospective or longitudinal design. Third, the measurement of fears was developed and used in the current study among FSWs in China. Further studies are needed to test validity and reliability of the measures. Forth, given the illegal and clandestine nature of commercial sex in China, our self-reported data are subject to socially desirable responses.

Despite of these limitations, our findings have several implications for HIV prevention and treatment service among low-paid FSWs. First, psychological fears can play an important role in HIV prevention social marketing or other public health campaigns. Strong fear may effectively motivate behavior change [34]. Many empirical studies in Africa suggest that the use of fear-based AIDS prevention campaigns often promote HIV-preventive behaviors [42]. For instance, Uganda's successful response to AIDS epidemic calls for attention to the fear appeal strategy: “Remarkably (Uganda) combined high fear approaches with openness and the capacity to rise above discrimination and to integrate prevention and care effectively” ([43], p.848).

Second, strengthening FSWs' trust in HIV-testing facilities and health care providers will be very important to promote FSWs' access to testing and other HIV prevention and treatment services. The severe stigma against both sex work and HIV/AIDS, the doubt about confidentiality of HIV clinics, and the distrust toward health care professionals or outreach workers may be sources of FSWs' fears of identity breaching and hesitation to access adequate and timely health care service [44]. In a study on utilization of VCT services among FSWs in northern China, 69% of the participants expressed their willingness to attend the VCT clinic, while only 11% were actually tested with fear of identity disclosure in the clinic being negatively related to actual HIV-testing [45]. Stigma reduction among health care professionals and secured HIV-testing results notification procedure with an emphasis on confidentiality protection are needed to decrease fear of identity breaching among FSWs. HIV education and prevention activities can be combined with other health care services that FSWs need (e.g., reproductive health, family planning and women health) to increase rapport and trust between FSWs and health care professionals [44].

Third, implementation of HIV prevention intervention needs multi-sector collaboration, including public health bureau, local authority and police. Although only a small number of FSWs had experience with law enforcement, fear of police arrests may amplify FSWs' reluctance to access HIV prevention services. It may be necessary to engage police in efforts to clarify some misunderstandings about HIV-clinics or VCT services (such as the misperception that HIV-testing records may be used by police to identify and detain FSWs) and reduce mistrust toward health care professionals.

In summary, our study has demonstrated how a psychological response among low-paid FSWs may be shaped by structural factors and how this psychological response may further influence their HIV-related behaviors. Our findings support the Social Cognitive Theory [46] which posits that socio-structural factors operate through psychological mechanism to produce behavioral effects. A growing literature has paid attention to the impact of psychological well-being on health behaviors among FSWs. For example, studies suggested that pessimism was associated with high risk sexual behaviors among FSWs [47], [48]. Fear of HIV infection may motivate FSWs to employ desirable HIV-preventive behaviors with high self-efficacy of protection. However, fears related to structural factors (e.g., policy, stigma) may result in negative outcomes (stress, depression, hopelessness), which may reduce their motivation to engage in protective behaviors, and weaken their motivation to adopt a long-term perspective on avoiding the negative consequences of risk behaviors [49], [50].

Fear is an emotion factor, but it may be intricately related to other cognition factors such as threat, self-efficacy, and peer norms. Further studies are needed to examine the interactions between fear and other psychological factors, and explore the mechanism of how fear affects health behaviors and mental health. Systematic studies on emotional context of FSWs will inform effective HIV prevention interventions among this high-risk population.
